# Editorial: The open challenges of cognitive frailty: risk factors, neuropsychological profiles and psychometric assessment for healthy aging

**DOI:** 10.3389/fnagi.2025.1679406

**Published:** 2025-08-19

**Authors:** Simone Varrasi, Sabrina Castellano, Daniele Magistro

**Affiliations:** ^1^Department of Educational Sciences, University of Catania, Catania, Italy; ^2^School of Health Sciences, University of Southampton, Southampton, United Kingdom

**Keywords:** cognitive frailty (CF), aging, health, quality of life, cognition

Cognitive frailty (CF), defined as the co-occurrence of physical frailty and mild cognitive impairment in the absence of dementia, has emerged as a critical concept in aging research. As populations grow older worldwide, there is increasing recognition that age-related decline is rarely confined to a single domain. Rather, it involves complex and overlapping deteriorations in physical health, cognitive function, psychosocial wellbeing, and functional capacity. Cognitive frailty captures this intersection, offering a holistic lens through which to understand vulnerability in later life. However, despite its growing prominence, cognitive frailty remains a contested and evolving construct. Key challenges persist regarding its operational definition, the selection of appropriate and sensitive assessment tools, and the identification of intervention strategies that are both scalable and person-centered. Existing approaches often rely on cognitive screening instruments or physical frailty models developed in isolation, which may not fully reflect the multidimensional nature of CF. Moreover, variation in the criteria used to diagnose CF across studies has made it difficult to build a coherent body of evidence and translate findings into practical guidance for clinicians or policymakers.

Recognizing these gaps, this Research Topic was conceived to bring together new research, methodological advancements, and interdisciplinary perspectives that can move the field forward. The Research Topic includes contributions from epidemiology, neuropsychology, gerontology, digital health, and rehabilitation sciences, offering both theoretical insights and applied findings. Specific areas addressed include the identification of modifiable risk factors, the development of functional and ecological assessments, the role of biomarkers and brain imaging, and the evaluation of technology-enhanced interventions.

Together, the articles in this Research Topic reflect a shared commitment to redefining cognitive frailty not only as a clinical condition, but as a dynamic and potentially reversible state. In doing so, they support a broader vision of healthy aging, one that prioritizes early detection, personalized care, and the integration of physical, cognitive, and social health domains.

## Beyond screening: functionally grounded assessment and neuropsychological profiling

While cognitive screening tools such as the MoCA are widely used, they may not capture functional impairments relevant to everyday living. Marks et al. demonstrate that performance-based assessments of instrumental activities of daily living (IADLs) identify limitations even among individuals who score within normative cognitive ranges, suggesting that a combined approach to assessment is essential for detecting subtle forms of CF.

## Consensus and co-creation in interventions

To address the absence of standardized intervention frameworks, Holland et al. convened a Delphi consensus panel involving clinicians, researchers, carers, and individuals with lived experience. The resulting 89 statements establish a foundation for evidence-based prevention and care strategies, highlighting the importance of participatory design in tackling complex aging syndromes.

## Psychosocial and sensory pathways to frailty

This Research Topic highlights how psychological distress and sensory loss intersect with frailty and cognitive decline. Mao et al. reveal that dual sensory impairment exerts its cognitive effects through anxiety and depression, moderated by frailty. Li et al. corroborate this by showing a robust link between chronic pain and CF. Additionally, Huang et al. find that short sleep duration is associated with increased frailty risk, reinforcing the role of lifestyle factors in CF onset.

## Sociodemographic disparities and health inequities

Several studies explore the structural drivers of CF. Zhang et al. report high prevalence rates of physical and cognitive impairment among older adults in southern China, with strong associations to income, rural residence, social support, and depressive symptoms. Ran et al. extend this analysis through joint longitudinal-survival models, showing that socioeconomic and behavioral risk factors, such as smoking and low education, exert persistent, and often sex-specific, effects on cognition. Mirkovic et al. similarly find that social support and educational level strongly correlate with memory performance among older adults in Serbia.

## Metabolic and somatic contributors to cognitive decline

The metabolic underpinnings of CF are becoming clearer. Luo et al. show that higher estimated glucose disposal rate (eGDR), a proxy for insulin sensitivity, is protective against cognitive decline, especially in women. Guo et al. identify an inverted U-shaped association between the Body Roundness Index and cognitive function, with mid-range values associated with the lowest risk, suggesting metabolic phenotype may serve as an early indicator of CF.

## Neurovascular, imaging, and biomarker innovations

Neurovascular integrity also appears relevant to CF. In Duan et al., extracranial–intracranial bypass surgery is associated with improved cognitive outcomes in elderly patients with cerebral steno-occlusive disease. Zhang et al. find that elevated serum levels of glial fibrillary acidic protein (GFAP) correlate with hippocampal subregion atrophy in individuals with MCI, proposing GFAP as a viable blood-based biomarker for early detection.

Further, Zhang et al. use Mendelian randomization to identify associations between frailty and cortical structure, while Chen et al. uncover shared genetic loci between body mass index and cognitive performance, highlighting *TUFM* and *MST1R* as potential targets for intervention.

## Post-stroke cognitive impairment and technological interventions

The lasting impact of stroke on cognitive health is reviewed in a bibliometric analysis by Zhang et al., which highlights cerebral small vessel disease as a major contributor to post-stroke cognitive impairment (PSCI). In an interventional study, Xue et al. show that ultrasound-guided stellate ganglion block reduces postoperative cognitive dysfunction by lowering inflammation and oxidative stress. In the realm of digital diagnostics, Tascedda et al. demonstrate the value of artificial intelligence, particularly graph neural networks, specifically Multi-Layer Perceptron, for differentiating Alzheimer's disease from MCI. Their work supports the growing use of AI in early and precise dementia diagnosis.

## Physical function, working memory, and daily functioning

Zhou et al. show a strong association between higher frailty index scores and reduced performance across multiple cognitive domains. Liao et al. further demonstrate that muscle strength influences working memory and, in turn, affects capacity for activities of daily living—supporting the rationale for integrated physical and cognitive interventions in older adults.

## Conclusion

This Research Topic brings into focus the multidimensionality and modifiability of cognitive frailty. Across biological, psychological, and social domains, the contributions highlight CF as a complex yet tractable condition, one that demands nuanced approaches to detection, prevention, and care. Together, these studies reinforce the view that cognitive frailty should not be regarded as an inevitable consequence of aging, but rather as a dynamic and potentially reversible state.

The Research Topic demonstrates the value of integrated assessment tools that move beyond screening and incorporate functional, neuropsychological, and ecological measures. It also underscores the importance of co-designed interventions that reflect the lived experiences of older adults, caregivers, and health professionals. Novel contributions from neuroimaging, biomarker research, and artificial intelligence point to promising frontiers in the early identification and personalized management of CF.

Equally, the findings call attention to persistent inequalities in access to healthcare, education, and social support that may shape the trajectories of frailty and cognitive decline. Addressing cognitive frailty, therefore, also requires attention to its social determinants, ensuring that future strategies are inclusive, equitable, and community-responsive.

Taken together, this Research Topic Collection offers a strong foundation for future work. It encourages the development of interdisciplinary frameworks and stakeholder-informed practices that can better support cognitive resilience in later life. As the field continues to evolve, efforts to clarify, operationalize, and address cognitive frailty will be essential to advancing the broader goal of healthy aging.

